# Mapped Clone and Functional Analysis of Leaf-Color Gene *Ygl7* in a Rice Hybrid (*Oryza sativa* L. ssp. *indica*)

**DOI:** 10.1371/journal.pone.0099564

**Published:** 2014-06-16

**Authors:** Xiao-juan Deng, Hai-qing Zhang, Yue Wang, Feng He, Jin-ling Liu, Xiao Xiao, Zhi-feng Shu, Wei Li, Guo-huai Wang, Guo-liang Wang

**Affiliations:** 1 College of Agronomy, Hunan Agricultural University, Changsha, Hunan, China; 2 Hunan Provincial Key Laboratory of Crop Germplasm Innovation and Utilization, Hunan Agricultural University, Changsha, Hunan, China; 3 State Key Laboratory of Hybrid Rice, Hunan, China; 4 State Key Laboratory for Biology of Plant Diseases and Insect Pests, Institute of Plant Protection, Chinese Academy of Agricultural Sciences, Beijing, China; 5 College of Plant Preservation, Hunan Agricultural University, Changsha, Hunan, China; 6 Department of Plant Pathology, Ohio State University, Columbus, Ohio, United States of America; National Taiwan University, Taiwan

## Abstract

Leaf-color is an effective marker to identify the hybridization of rice. Leaf-color related genes function in chloroplast development and the photosynthetic pigment biosynthesis of higher plants. The *ygl7* (*yellow-green leaf 7*) is a mutant with spontaneous yellow-green leaf phenotype across the whole lifespan but with no change to its yield traits. We cloned gene *Ygl7* (Os03g59640) which encodes a magnesium-chelatase ChlD protein. Expression of *ygl7* turns green-leaves to yellow, whereas RNAi-mediated silence of *Ygl7* causes a lethal phenotype of the transgenic plants. This indicates the importance of the gene for rice plant. On the other hand, it corroborates that *ygl7* is a non-null mutants. The content of photosynthetic pigment is lower in *Ygl7* than the wild type, but its light efficiency was comparatively high. All these results indicated that the mutational YGL7 protein does not cause a complete loss of original function but instead acts as a new protein performing a new function. This new function partially includes its preceding function and possesses an additional feature to promote photosynthesis. *Chl1*, *Ygl98*, and *Ygl3* are three alleles of the *OsChlD* gene that have been documented previously. However, mutational sites of *OsChlD* mutant gene and their encoded protein products were different in the three mutants. The three mutants have suppressed grain output. In our experiment, plant materials of three mutants (*ygl7*, *chl1*, and *ygl98*) all exhibited mutational leaf-color during the whole growth period. This result was somewhat different from previous studies. We used *ygl7* as female crossed with *chl1* and *ygl98*, respectively. Both the F_1_ and F_2_ generation display yellow-green leaf phenotype with their chlorophyll and carotenoid content falling between the values of their parents. Moreover, we noted an important phenomenon: *ygl7*-NIL's leaf-color is yellow, not yellowy-green, and this is also true of all back-crossed offspring with *ygl7*.

## Introduction

Genetic purity of crop plants, which has a critical impact on increasing crop yields, is gradually becoming the focus in hybrid rice production [1]. Molecular markers can be used to rapidly identify seed purity yet are not useful for identifying off-type plants. Plants with a distinct color phenotype can be easily identified and removed, so leaf-color has become a suitable marker for maintaining the genetic purity of hybrid rice [2]. In addition, study of the genetic mechanism of leaf-color mutations would further our understanding of chlorophyll biosynthesis and degradation, chloroplast development, tetrapyrrole synthesis, and photosynthesis.

The colors of leaf-color mutants include albino, chlorosis, thermo-color, light green, maintaining green, stripes and zebra, green-revertible albino, dark-green, and purple [3]. As of 2013, at least 208 leaf-color mutants have been identified in rice. Of those, 175 mutants have been analyzed and 154 genes have been mapped to all 12 chromosomes. However, only 53 leaf-color genes of rice have been cloned (14 of them had alleles). Among them, 14 genes function directly in chlorophyll biosynthesis and catabolism (DCBC) [4]–[11], while 6 genes indirectly take part in those two processes (ICBC) [12]–[15]. Sixteen genes are directly involved in the developmental regulation of chloroplast (DDC) [16]–[21], while another 3 genes are involved indirectly (IDC) [22]. Four genes take part in the metabolism of carotenoids (CBM) [23]–[26]. One gene influences anthocyanin biosynthesis and catabolism (ABC). Three genes influence rice leaf-color via other pathways (OP) [27]. The functions of the remaining 6 genes are not yet defined (NDF) [28]–[32]. Details are provided in Table S1.

Mg-chelatase, which was first discovered in photosynthetic bacteria, is the first enzyme catalyzing the committed step of chlorophyll synthesis. Mg-chelatase consists of three subunits: I (40-kDa), D (70-kDa), and H (140-kDa) [33]. Only one copy of all the three subunits exists in rice. However, there is more than one subunit I in some species, such as Arabidopsis [34]. Mg-chelatase activity can only be detected when all three subunits are present [35]. Subunit I of Mg-chelatase belongs to the functionally diverse superfamily of AAA proteins and has the ATPase activity which is associated with various cellular activities [36]. Subunit D of Mg-chelatase has three consecutive regions: an N-terminal domain homologous to the AAA module of subunit I but without ATPase activity, a central acidic and Pro-rich region, and an integrin I domain with an unknown function at the C-terminus [37]. ATP combines with subunit I, subunit D, and Mg^2+^ to form the I-D-Mg-ATP complex. Subunit H combines with Protoporphyrin IX to form the H- Protoporphyrin IX complex. Then, I-D-Mg-ATP and H-protoporphyrin IX are combined together to hydrolyze ATP. Simultaneously, Mg^2+^ is added to Protoporphyrin IX to form Mg-Protoporphyrin IX [38].

Using the map-based cloning method and T-DNA insertion mutants, the five genes *Chl1* [39], *Chl9* [39], *Ygl9* [40], *Ygl3* [9], and *ChlH* [41] have been cloned in rice. *Chl1*, *Ygl98*, and *Ygl3* code for OsChlD, and they are alleles. *Chl9* codes for OsChlI and *ChlH* codes for OsChlH. Plants with *ch11* exhibit yellowish-green leaves at the seedling stage, and then the color turns to a normal green at maturity. The mutational allele of *Chl1* has a single base change from G to A at position 1176 bp which is located at the 8^th^ exon. This nucleotide change leads to one aa conversion from Arg (R) to Gln (Q) at position 393 aa [39]. The *ygl98* mutant exhibits a yellow-green leaf phenotype throughout the growing season and lower grain output. Mutational *Yg198* has a single base change from G to A at position 1522 bp which is located at the 10^th^ exon. This conversion results in an amino acid change from Ala (A) to Thr (T) at position 508 aa [40]. The *ygl3* mutant displays a yellowy-green leaf-color throughout the growth cycle, lower plant height, and lower grain yield. The mutational *Yg13* has a single base change from G to C at position 1009 bp which is located at 6^th^ exon. This mutation leads to an amino acid change from Ala (A) to Thr (T) at position 337 aa [9].

The rice variety An Nong Biao 810S is a natural, yellowy-green leaf mutant selected from the PTGMS (photo- and thermo-sensitive genetic male sterile) rice line An Nong 810S maintained by Huaihua Vocational and Technical College of Hunan [42]. This plant leaf displays a permanent yellowy-green color throughout its life. Several studies reported no remarkable differences between wild type and the mutant in other primary agronomic traits [43], [44]. Compared to the wild type An Nong 810S, the mutant has a sharp drop in chlorophyll content. This variety as a visual marker is useful for testing the varietal purity of hybrid rice seeds. The leaf-color phenotype in this mutant is controlled by the single recessive nuclear gene *Ygl7* which encodes a magnesium-chelatase ChlD. In this study, a single-nucleotide mutation from T to C at position 1883 bp of gene *Ygl7* which leads to a change in the protein (Leu to Ser at position 628 aa)is identified by genetic sequencing. The RNAi assay of normal *Ygl7* has been conducted in Nipponbare. Interestingly, back-crosses between the mutant *ygl7* and other varieties displayed yellow-leaves. This research is significant for the application of a leaf-color marker gene in hybrid rice production and for exploration of the chlorophyll synthesis mechanism.

## Materials and Methods

### Plant materials

These experiments were conducted at Hunan Changsha and Hainan Sanya in April 2009 to October 2013. We used the following varieties.

The mutant *ygl7* was a spontaneous yellow-green leaf mutant derived from multiplication of the PTGMS rice variety An Nong 810S (810S) (*Oryza sativa* L. ssp. *indica*) [42]. For genetic analysis, we used three combinations derived from crossing *ygl7* with two *indica* cultivars (353 and 9311) and a *japonica* variety termed Nipponbare (NPB). The F_2_ population derived from crossing *ygl7* with NPB was used to map *Ygl7*. The *ygl7*'s near-isogenic line *ygl7*-NIL was used as a genetic complement. We constructed the near -isogenic line *ygl7*-NIL to function as acceptor material of the genetic transformation. Nipponbare was the acceptor parent back-crossed with the donor parent, *ygl7*. The yellow plants' population from BC_4_F_2_ is *ygl7*-NIL. The *Chl1* mutant (obtained from Professor Nam-Chon Paek, College of Agriculture and Life Sciences, Seoul National University) and the *ygl98* mutant (obtained from Professor Wang Ping-rong, Sichuan Agriculture University) are the *OsChlD* allele's mutant. All plant materials were planted under a standard management.

### Genetic analysis and mapping

The leaf-color phenotype in the F_1_ and F_2_ populations were designated as the wild type (normal green leaf) and the yellowy-green leaf phenotype was considered the mutant. The F_2_ segregation ratios were analyzed with a χ^2^ goodness of fit test using the Excel software.

DNA was extracted from leaves using the improved CTAB method [45]. A total of 1199 molecular markers were used in this study, including 1130 SSRs (simple sequence repeat), 67 SFPs (single feature polymorphism), and 2 STSs (sequence tagged site). The SSR markers were obtained from Gramene (http://www.gramene.org/microsat/). The SFP and STS markers were designed using the Primer 5.0 software (Table S2). First, we used 1100 SSRs and 67 SFPs to select polymorphic markers from *Ygl7* and NPB. Then, we used the entire gene pool to obtain potential linked markers. We used those markers in primary mapping of F_2_ individuals with the yellowy-green leaf phenotype that were selected from the cross of *Ygl7* and NPB. To fine map the *Ygl7* locus, we selected an additional 30 SSRs, 4 SNPs, and 2 STSs. Six molecular markers were used to screen recombination events from 2,849 F_2_ individuals for fine-mapping. Linkage analysis was conducted with MAPMAKER3.0, and the linkage map was constructed with MapDraw V2.1.

### Sequence analysis

Within the fine mapped chromosome region, candidate genes were screened according to the annotation database of the NCBI. Specific primers were designed according to the genome sequences of NPB. These primers were used to amplify the candidate genes from the *ygl7* mutant and its wild type parent 810S. The amplified products from DNA and RNA were sequenced and structurally analyzed to determine the target gene and the mutation site within the target gene. Total RNA was extracted using the TRIZOL method [46].

### Genetic complementation and RNAi suppression of *YGL7*

The PCR products of a full-length *YGL7* cDNA (primer D-18, Table S2) were digested with *BamH*I and *Kpn*I and then inserted into the vector pLYL18, which was derived from pCAMBIA1300 using an ubiquitin promoter (Figure S1). Because callus of the *ygl7* mutant (an indica variety) was difficult to obtain, the recombinant plasmid designated as pLYL18-YGL7 was introduced into Agrobacterium tumefaciens EHA105. It was then used to infect calli of individuals with the homozygous *ygl7* allele in japonica genetic background selected from one NPB/*ygl7* BC_4_F_2_ population (*ygl7*-NIL). Transformation was conducted according to a published protocol [47].

The pFGC5941 with a 35S promoter and a petunia CHSA (chalconesynthase A) intron was used as an RNAi vector. The PCR product is 592 bp conserved segments of cDNA *YGL7* (primers DXJRNAi II, Table S2). The sense segment link to pFGC5941 was accomplished using *Nco*I and *Asc*I, and *BamH*I and *Xba*I were used to link the anti-sense segment (Figure S1), which was then transformed into NPB as described above.

### Gene Expression Analysis

For quantitative real-time PCR (qRT-PCR) analysis, total RNA was extracted from young leaves of *ygl7*, 810S and *ygl7*-NIL using an RNA Prep Pure Plant kit (Tiangen Co., Beijing, China). RNA was reverse transcribed using a SuperScript II kit (TaKaRa). Real-time PCR was performed using a SYBR Premix Ex Taq™ kit (TaKaRa) on an ABI prism 7900 Real-Time PCR System. We selected two types of genes for analysis. The first type are associated with chlorophyll biosynthesis and includes ChlD (YGL7), ChlI, ChlH, YGL1, HEMA1, and PoPA [32]. The second type are associated with photosynthesis, including the genes Cab1R, Cab2R, PsaA, PsbA, and RbcL [32]. qRT-PCR primers come from Zhou KN's paper [32] (primers were DXJCHLD to DXJrbcL, Table S2).

### Chlorophyll fluorescence and photosynthetic pigments

Chlorophyll fluorescence was determined through IMAGING-PAM at the early heading stage. Fo, Fv'/Fm', ETR, qN, and qP were used in our study.

The chlorophyll (Chl) and carotenoid (Car) contents were measured using a spectrophotometer according to the method described by Tang Yan-lin [48]. To summarize briefly, equal weights of freshly collected second top leaves from the heading stage were immersed in extracting solution for 12 h under dark conditions. The extraction solution had a volume ratio of acetone, ethanol, and distilled water of 4.5: 4.5: 1. Residual plant debris was removed by centrifugation. The supernatants were analyzed with a DU 1700 UV/Vis Spectrophotometer at 440 nm, 663 nm, and 645 nm using the following equations:

Chla(mg/g) = [(9.784OD663-0.990OD645)×V]/(M×1000)

Chlb(mg/g) = [(21.426OD645-4.650OD663)×V]/(M×1000)

Chl(mg/g) = [(5.134OD663+20.436OD645)×V]/(M×1000)

Car(mg/g) = [4.695OD440-0.268(Chla+Chlb)×V]/(M×1000)

## Results

### Characterization of the *ygl7* mutant and *ygl7*-NIL

**Figure 1:**
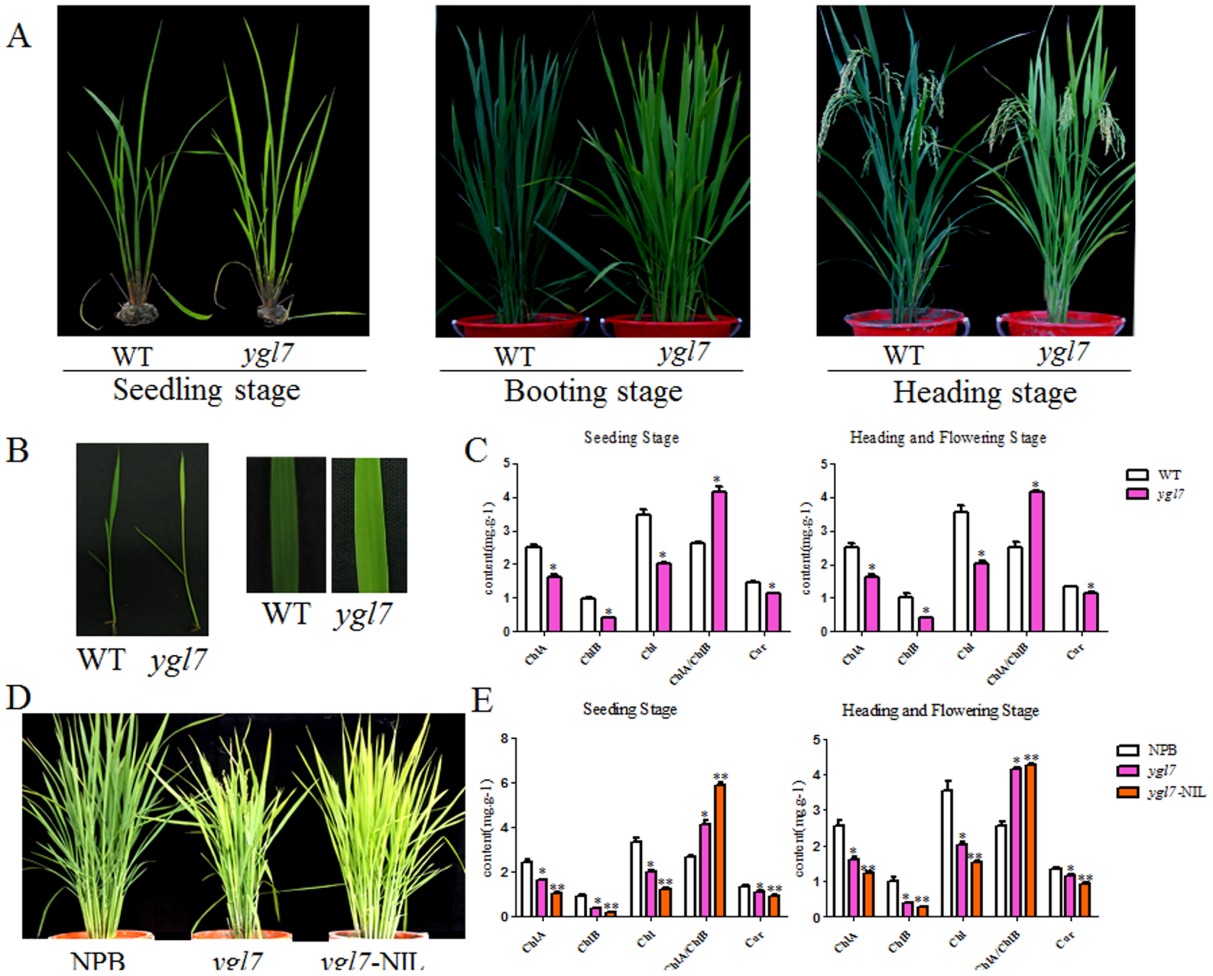
Characterization of *ygl7* and *ygl7*-NIL phenotypes and leaf pigments. A. Phenotypes of wild type (WT) and *ygl7* at seedling, booting, and heading stages. B. Leaf-color comparison between WT and *ygl7*. C. Leaf pigment contents of WT and *ygl7* at booting and heading stages. D. Phenotypes of *ygl7*-NIL and its parents, NPB (acceptor) and *ygl7* (donor). E. Comparison of leaf pigments contents of *ygl7*-NIL with its parents, NPB and *ygl7* at booting and heading stages. Values are the mean ± SD of three replicates.

The rice mutant *ygl7* has a yellowy-green leaf under paddy field conditions; *ygl7* exhibited a distinct and steady phenotype throughout its lifespan when grown in Hunan Province and Hainan Province from 2009 to 2013. The *ygl7* plants showed an enduring yellow-green leaf phenotype that began when the first leaf completely unfolded (Figure 1 A, B). This color extended to the spikes, stalks, and other above-ground plant parts. Compared to 810S, there were no significant differences in main agronomic traits including the growth period, plant height, spike length, the spike number per plant, the grain number per spike, effective tiller, maturing rate, and 1000-grain weight (Table 1). Compared to same combinations which crossed with 810S, there were no significant differences in main agronomic traits, including the growth period, maturing rate, and 1000-grain weight (Table 2). These results suggested that this phenotype does not influence grain output and hybrid characteristics. The contents of Chl a, Chl b, and carotenoids were significantly reduced in *ygl7* by about 50% compared to the wild-type parent 810S (Figure 1 C). These results suggested that the *ygl7* mutant phenotype mainly resulted from reduced contents of photosynthetic pigments. In addition, the Chl a/b ratio of *ygl7* increased compared to wild type, which indicated that the drop of Chl b was greater than Chl a in the *ygl7* mutant.

**Table 1:** The main agronomic traits of *ygl7*'s propagation.

Varieties	Growth period (d)	Plant height (cm)	Spike length(cm)	The spike number per plant	The grain number per spike	Effective tiller (branch)	Seed setting rate (%)	1000 - grain weight (g)
*ygl7*	69±4A	84.3±3.4A	19.8±1.0A	11.2±0.6A	92.4±3.1A	11.0±4.0A	61.3±3.6A	25.1±1.1A
810S	68±4A	85.6±3.0A	19.6±0.8A	10.9±0.7A	90.9±2.2A	10.2±3.0A	60.7±4.5A	24.6±0.8A

**Table 2:** F_1_ agronomic traits.

Parents		Growth period (d)	Spike length(cm)	The spike number per plant	The grain number per spike	F_1_ seed setting rate (%)	F_1_ 1000 - grain weight (g)
Female	Male						
*ygl7*	9311	89±4B	18.8±0.8B	11.7±2.2B	96.3±1.2B	77.5±5.4B	25.7±0.7B
810S	9311	90±4B	19.0±1.1B	10.9±0.8B	95.2±1.2B	76.7±6.2B	25.3±0.6B
*ygl7*	NPB	70±4A	17.9±0.9A	11.5±2.0A	101.1±3.0B	66.7±3.7A	23.9±0.5A
810S	NPB	72±3A	17.8±0.7A	11.1±1.7A	99.3±2.8A	65.2±4.1A	23.5±0.7A

The *ygl7*-NIL is the *ygl7*'s near isogenic line. To construct *ygl7*-NIL, the donor parent *ygl7* was back-crossed with the receptor parent NPB. We found that *ygl7*-NIL displayed an abnormal leaf-color phenotype. The leaf-color of *ygl7*-NIL is yellow instead of yellow-green (Figure 1 D). The contents of Chl a, Chl b, and carotenoids were reduced in *ygl7*-NIL compared with *ygl7* (Figure 1 E). The same phenomenon was observed when *ygl7* was back-crossed with indica rice (data not shown). The situation is probably caused by an altered genetic background. It is also probably caused by the mutation of how YGL7 joins in other regulatory pathways. This phenotypic characterization improves the ability to cultivate better sterile lines and will facilitate hybrid seed production and purification for the growth of rice crops.

### Single amino acid change in *Ygl7*

All F_1_ plants of the *ygl7* mutant crossed with normal green rice varieties displayed normal green leaves. The F_2_ populations from *ygl7*/9311, *ygl7*/353, and *ygl7*/NPB showed a segregation ratio of 3∶1 (green: yellow-green plants, χ^2^<χ^2^_0.05_ = 3.84, P>0.05; Table 3). These data indicated that the yellow-green leaf phenotype in the *ygl7* mutant was controlled by a single recessive nuclear gene.

**Table 3:** Segregation of F_1_ and F_2_ populations from three crosses.

Combination	F_1_ population	F_2_ population				
		No. of yellowy- green plants	No. of green plants	ratio	χ^2^	P value
*ygl7*/353	green	425	1326	1∶3.12	0.03	0.9
*ygl7*/9311	green	642	1912	1∶2.98	0.02	0.9–0.75
*ygl7*/NPB	green	724	2125	1∶2.94	0.08	0.9–0.75

**Figure 2:**
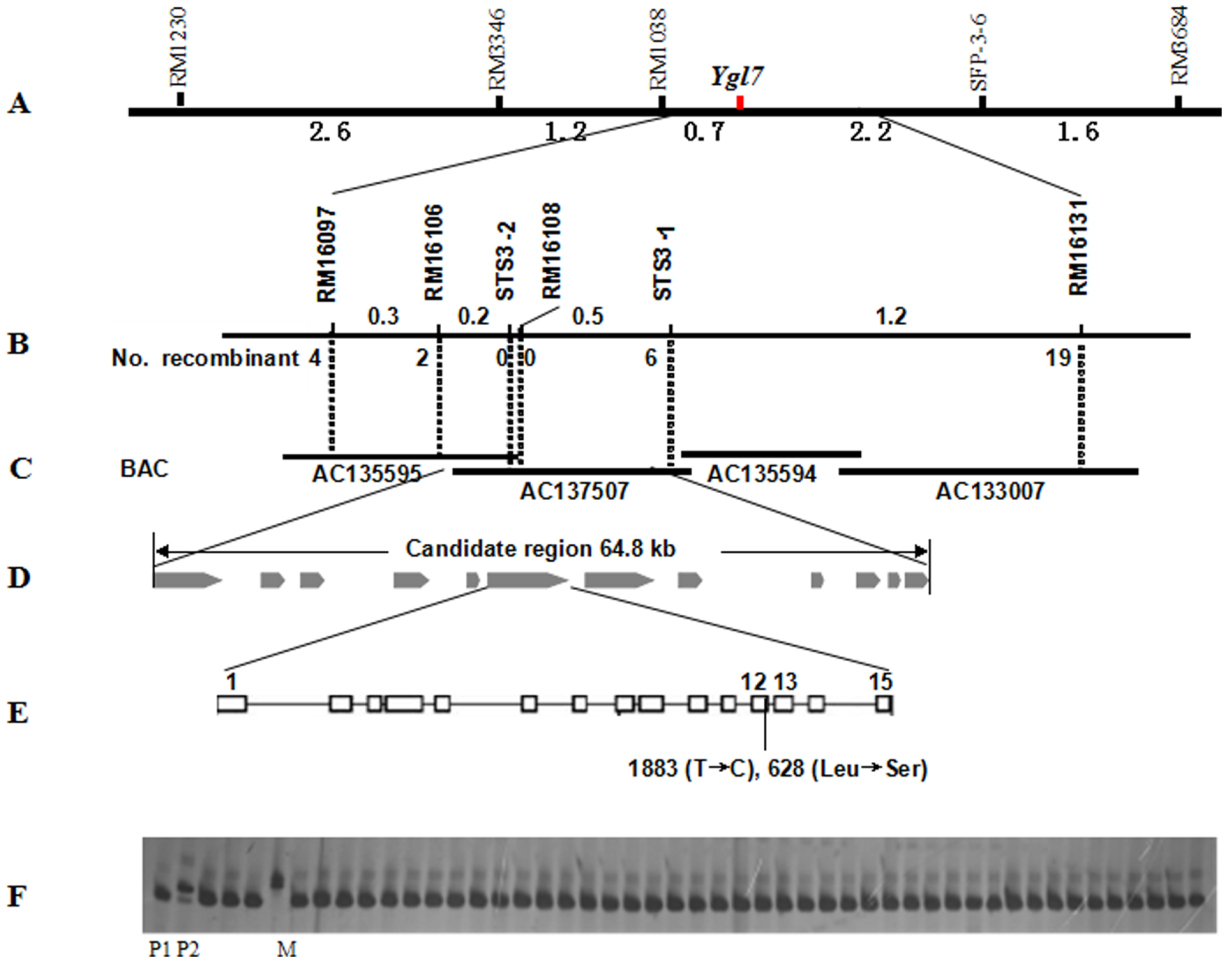
Map-based cloning of the *Ygl7* locus. A. Preliminary map of the *Ygl7* locus between RM1038 and SFP-3-6 based on analysis of 368 F_2_ (*ygl7*/NPB) individuals. Numbers below indicate the genetic distance (cM) between the markers. B. Fine mapping of the *Ygl7* locus, which was narrowed down to a 64.8 kb genomic DNA region between RM10106 (2 recombinants) and STS3-1 (6 recombinants) based on 651 F_2_ individuals. C. *Ygl7* locus was covered by two BAC contigs AC135595 and AC137507. D. There were 12 expressed genes identified in the fine mapping of *Ygl7*'s area. E. One candidate *Ygl7* gene (Os03g59640) was found to contain one base replacement (1883, T→C) resulting in one amino acid mutation (628, Leu→Ser). F. PCR analysis of the F_2_ yellowy-green leaf plants from *Ygl7* crossed with NPB. Marker, RM16108; P1, *Ygl7*; P2, NPB; M, 100 bp low ladder; others are individual yellowy-green leaf plants of the F_2_ from *Ygl7* crossed with NPB.

Locus mapping of the *Ygl7* gene was performed using the F_2_ population from a *ygl7*×NPB mutant cross. A Bulked Segregant Analysis (BSA) suggested that the *Ygl7* locus was possibly located on chromosome 3 or 12 (Table S3). The *Ygl7* locus was initially mapped to within 3.9 cM between RM1308 and SFP-3-6 on the long arm of chromosome 3 based on 368 typical yellow-green leaf F_2_ individuals (Figure 2 A). With a total of 651 F_2_ homozygous mutant plants, we fine-mapped the *Ygl7* locus to a 64.8 kb interval between SSR marker RM16106 and STS marker STS3-1 on BAC clones AC135595 and AC137507 (Figure 2 B, C). We found 12 ORFs were predicted within this region (Figure 2 D, Table S4). Among these ORFs, LOC_Os03g59640 was highly correlated with the *ygl7* phenotype. The LOC_Os03g59640 ORF encodes the subunit CHLD of magnesium-chelatase. Comparison of sequences indicated that the single base change (T1883C) located in the 12^th^ exon of Os03g59640 resulted in a missense mutation (L628S) in the encoded product (Figure 2 E, F). The cDNA sequenced from Os03g59640's was the same missense mutation as the sequenced DNA. For these reasons, this gene was identified as the candidate gene of *Ygl7* that caused the phenotype of yellow-green leaves during the whole growth stage.

### Confirmation of *Ygl7* function

**Figure 3:**
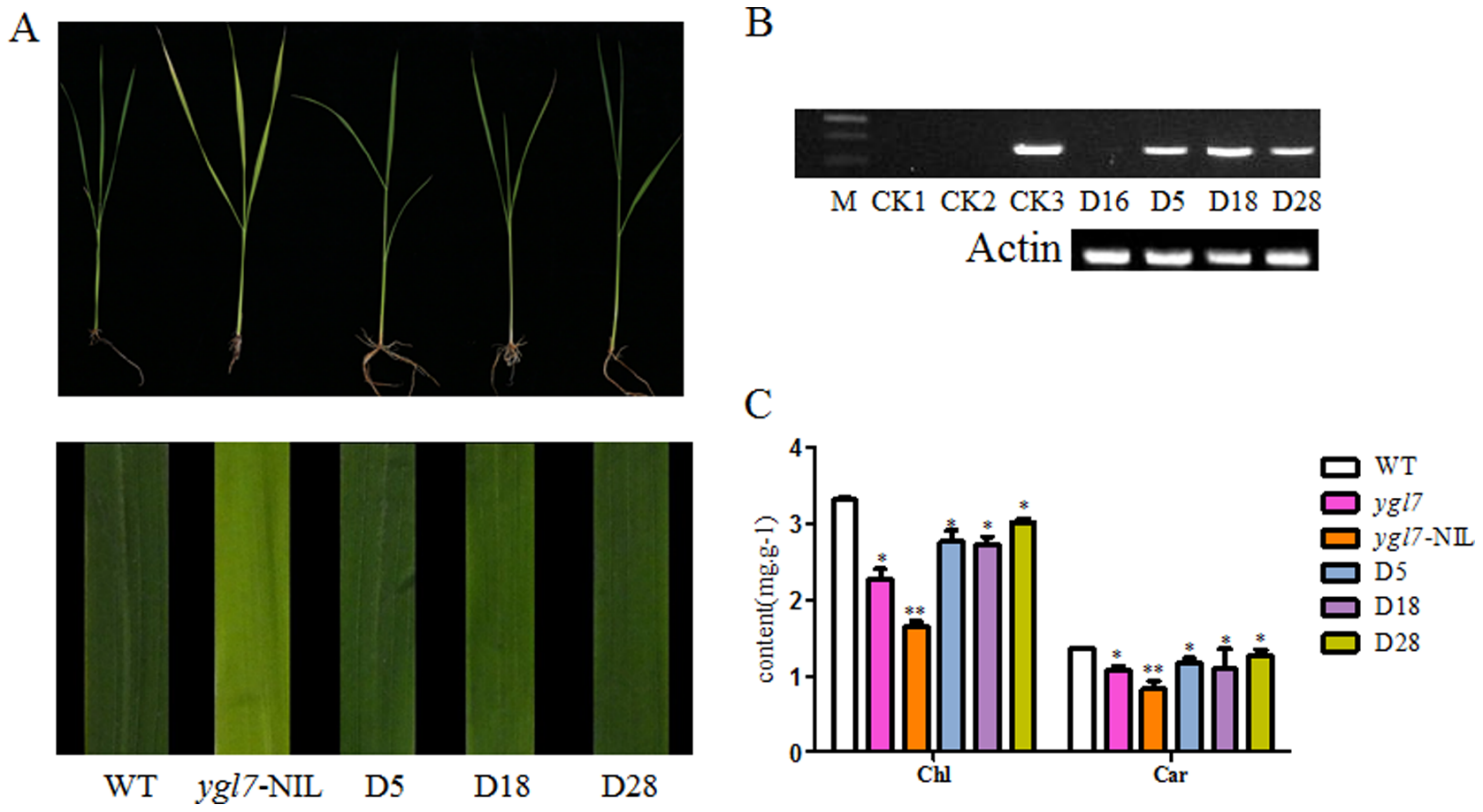
Expression analysis of *Ygl7*. A. Leaf phenotypes in the complementation test. B. The PCR detection of HPT in some transgenic lines. CK1, H2O; CK2, normal plant of NPB; CK3, plasmid pLYL18; D16, the transgenic plant without the *YGL7* gene; D5, D18, and D28 are the transgenic plants with the *YGL7* gene. C. Pigment contents. Values are the mean ± SD of three replicates.

To confirm whether the single base change in *Ygl7* is responsible for the mutant phenotype, we performed a complementation analysis. Complementation analysis was performed by transforming *ygl7*-NIL with the *YGL*-cDNA transgene in pLYL18. The plasmids with *YGL7* contained the bacterial hygromycin B phosphotransferase (HptII) gene as a selection marker (Figure 3 B). The positive transgenic plants were almost reverted to green leaf and their Chls and Cars contents approached the levels of wild type plants (Figure 3 A, C).

**Figure 4:**
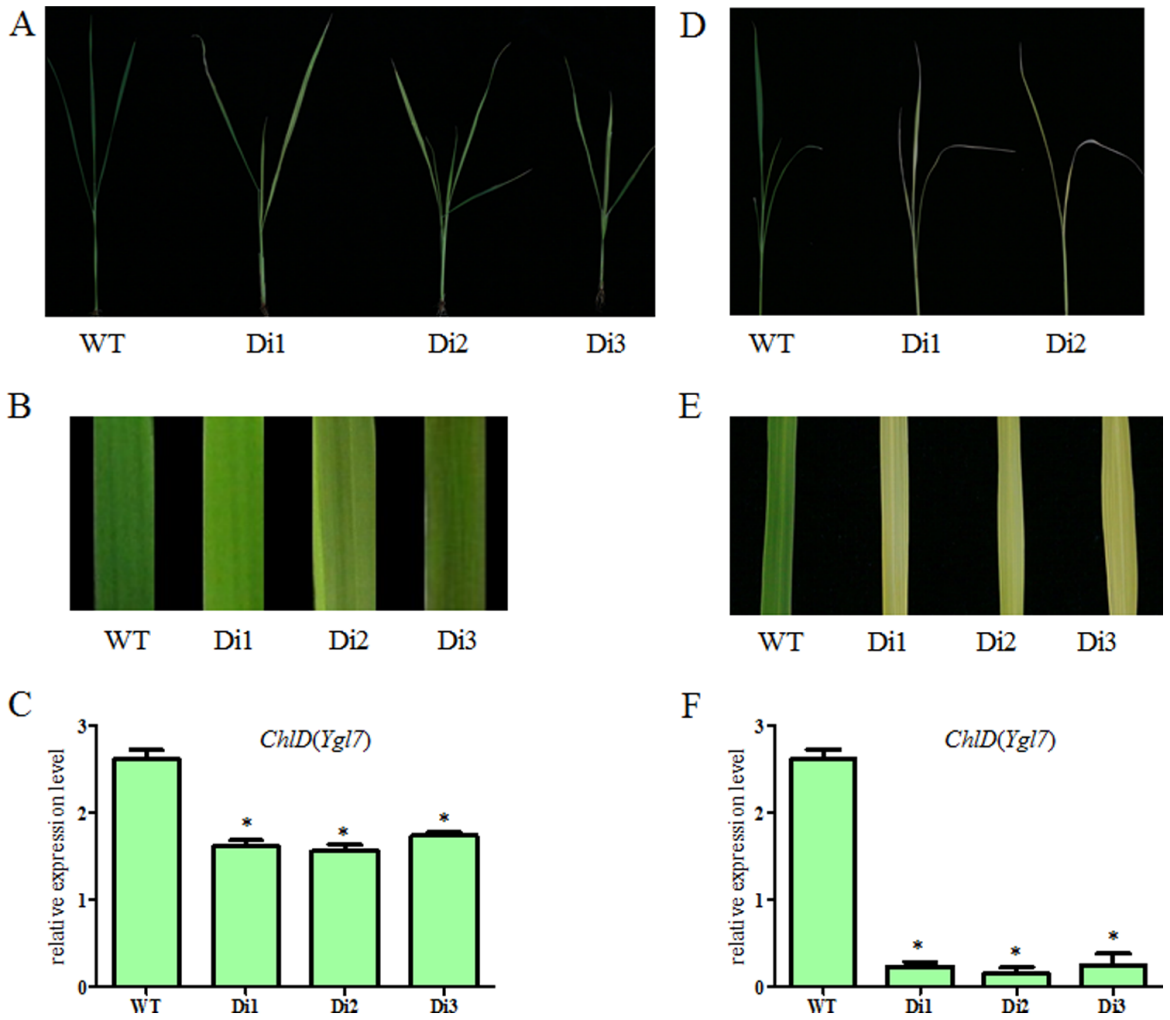
RNAi analysis of *Ygl7*. A. and B. Phenotypes of leaf in the RNAi plants at 5 leaves. C. The expressions of *ChlD* (*Ygl7*) genes in tRNAi plants at 5 leaves. D. and E. Phenotypes of leaf in the RNAi plants at 7 leaves; old leaves were dead. F. The expressions of *ChlD* (*Ygl7*) genes in the RNAi plants at 7 leaves. Values are the mean ± SD of three replicates. Di1, Di2 and Di3 are the RNAi plants.

RNA interference was conducted by transforming NPB with a 594 bp conservative segment of cDNA transgene in pFGC5941. A total of 9 independent transgenic lines were obtained. The first three leaves of RNAi transformed plants were green, but over time all leaves gradually turned from pale-green (Figure 4 A, B) to yellowy-green, then to yellow, then to almost white, and then died (with about seven-to-eight leaves) (Figure 4 D, E). This data suggested that RNAi-mediated silencing of *YGL7* causes a lethal phenotype of in the transgenic plants. Real-time PCR analysis showed that the magnitude of *YGL7* expression in the transgenic lines was reduced compared to the NPB (Figure 4 C, F).

These results demonstrate that Os03g59640 corresponds to the *Ygl7* gene. Moreover, silencing *Ygl7* is a gradual process. Once the silencing is completely, rice is unable to synthesize chlorophyll which leads to the death of the plant. The combination of RNAi and complementation analysis shows that the mutation of YGL7 does not cause a loss of OsChlD's functions. The mutation instead endows the protein with a new function.

### The *ygl7* mutant uses light energy efficiently

**Figure 5:**
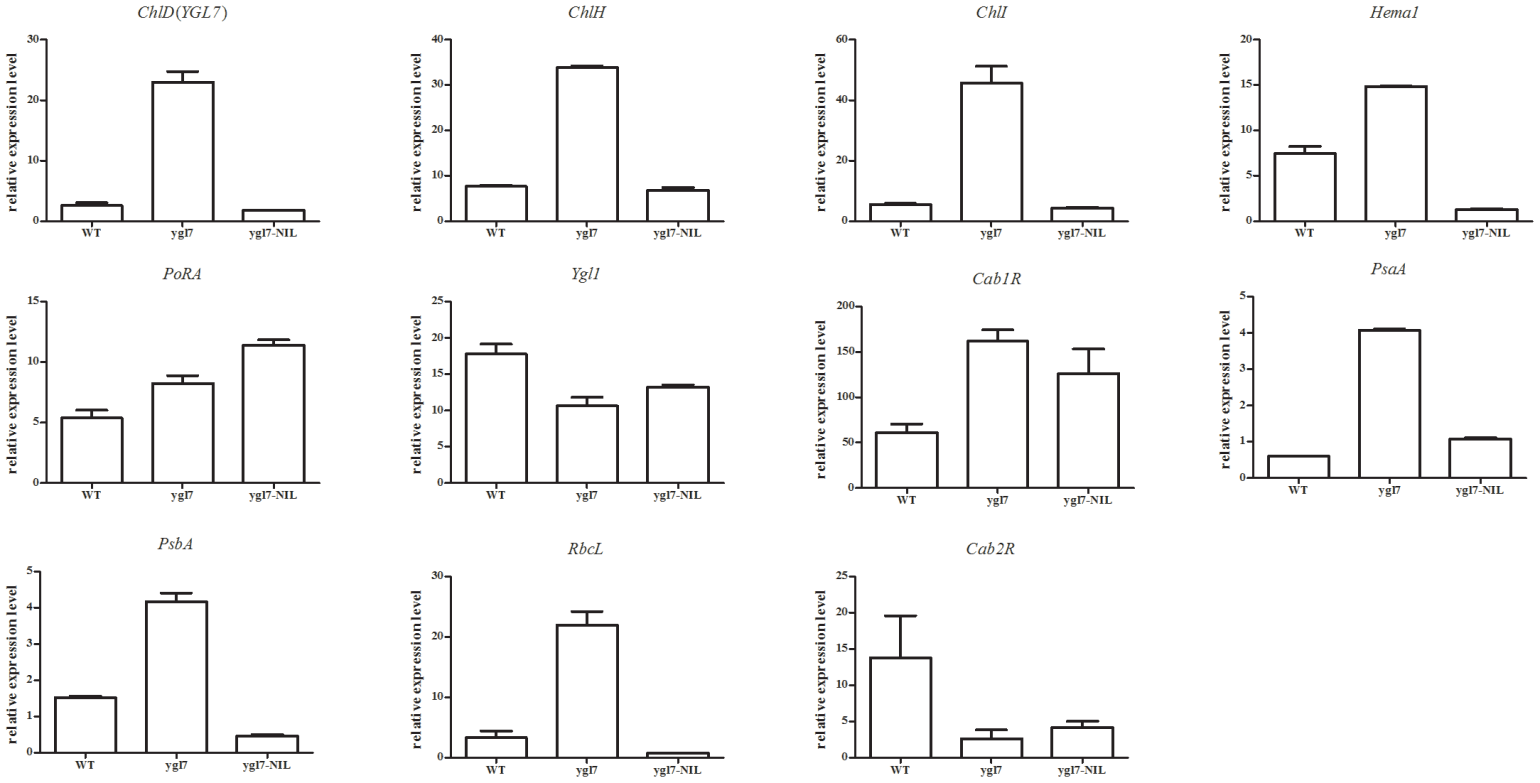
Real-time PCR expression analysis of genes associated with chlorophyll biosynthesis and photosynthesis in WT and *ygl7*. Genes involved in chlorophyll biosynthesis included *ChlD*(*YGL7*), *ChlI*, *ChlH*, *Hema1*, *PoRA,* and *Ygl1*. Genes involved in photosynthesis included *Cab1R*, *Cab2R*, *PsaA*, *PsbA*, and *RbcL*. Values are the mean ± SD of three replicates.

Real-time PCR was performed to study the expression profile of *ygl7*. In *ygl7*, *ChlD*, *ChlI*, and *ChlH* (Mg-chelatase D, I and H subunit) are up-regulated compared in the *ygl7* to the WT (810S). However, *ChlD* was much lower in *ygl7* than *ChlI* and *ChlH* (Figure 5). This illustrates that the mutation *ChlD* led to an accumulation of the three subunits of Mg chelatase, especially *ChlI* and *ChlH*. This shows that the yellow-green leaf resulted from unusual chlorophyll biosynthesis controlled by the accumulation of *ChlD*, *ChlI*, and *ChlH*.

Real-time PCR was performed to determine whether the *Ygl7* gene has any relationship with other genes and to determine what kind of genes are related to *Ygl7*. Those genes associated with chlorophyll biosynthesis (including *ChlD*, *ChlI*, and *ChlH*), *Hema1* (glutamyl tRNA reductase), and *PoPA* (NADPH-dependent protochlorophyllide oxidoreductase) were up-regulated in *ygl7* compared with wild type. Similarly, those genes associated with photosynthesis, including *Cab1R* (light-harvesting Chl a/b-binding proteins of PSII), *PsaA* (reaction center polypeptides of PSI), *PsbA* (reaction center polypeptides of PSII), and *RbcL* (the large subunit of Rubisco), were also mostly up-regulated in *ygl7*. However, *Ygl1* (Chl synthetase) and *Cab2R* (light-harvesting Chl a/b-binding proteins of PSII) were down-regulated in the *ygl7* mutant (Figure 5). Thus, it seems that the *ygl7* mutant affects transcription of not only the *Ygl7* gene itself but also of genes associated with Chl biosynthesis and photosynthesis. The normal accumulation of *ChlD* might attract accumulation of other Chl biosynthesis-related genes that then destroys the chlorophyll biosynthetic pathway.

Interestingly, expression of these genes in *Ygl7*-NIL was at almost normal levels (Figure 5). Only a few Chl biosynthesis-related genes and photosynthesis-related genes were affected by *Ygl7*. We have no plausible explanation for this at present.

**Figure 6:**
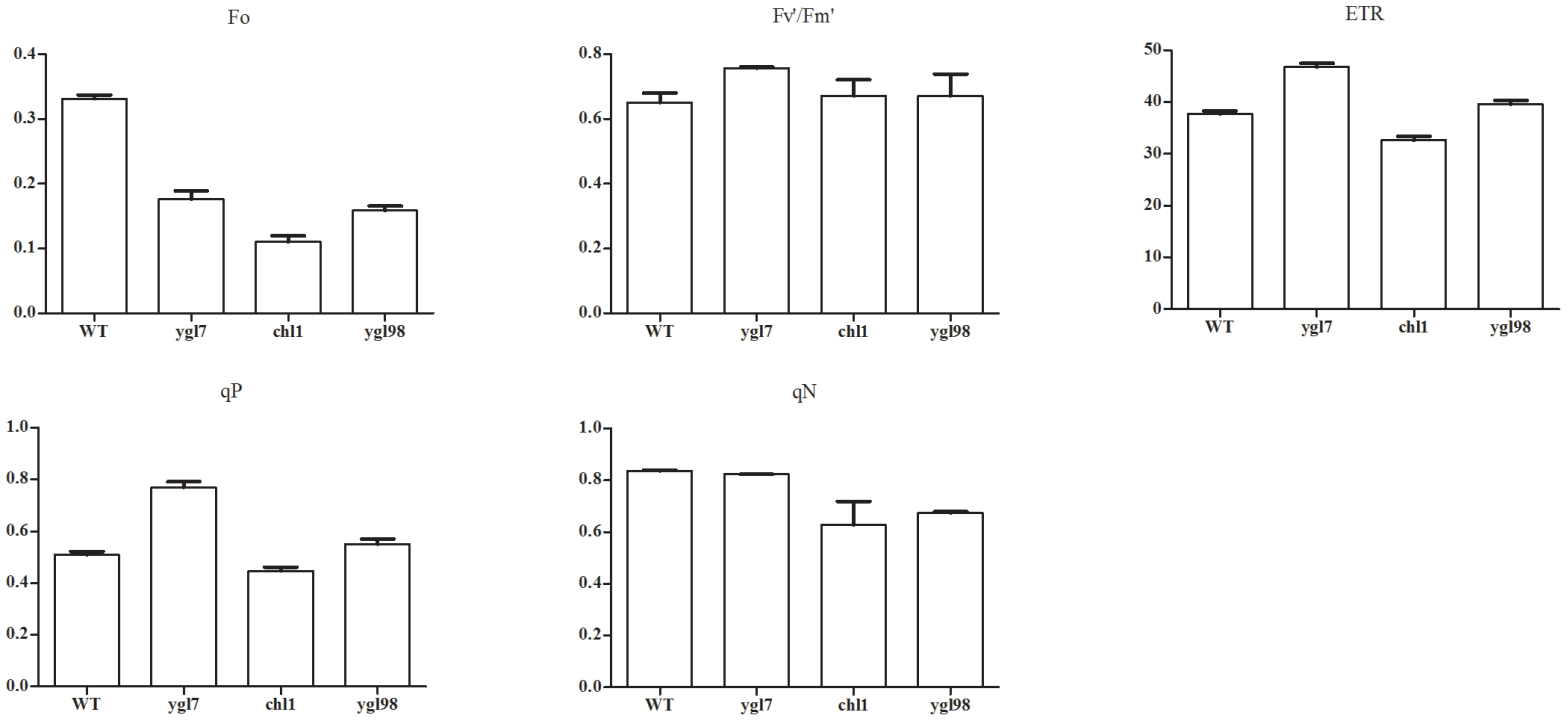
Chlorophyll Fluorescence in three leaf-color mutants. Fo: immobilized fluorescent, Fo coincides chlorophyll content; Fv'/Fm': effective photochemical quantum yield from PSII; ETR: the electron-transfer; qP: photochemistry quenching; qN: non-photochemistry quenching.

We performed Chlorophyll Fluorescence in *ygl7*, *chl1*, and *ygl98*. The Fo (minimal fluorescence) values from this chlorophyll fluorescence were consistent with the leaf chlorophyll content in *ygl7*, *chl*, and *ygl98* (Figure 6). The data illustrated that the chlorophyll fluorescence detector is trustworthy. Fv'/Fm' (photochemical efficiency of PSII in the light), ETR (photosynthetic electron transport rate), and qP (photochemical quenching coefficient) were higher in *ygl7* than WT, *chl1*, and *ygl3* (Figure 6). This indicated that conversion efficiency of light energy and capture efficiency of solar energy in the PSII center is higher in *ygl7*. The transport number of photosynthetic quantum yield and the transport rate of acyclic electron from antenna pigments in PSII were also higher in *ygl7*. qN is a coefficient of non-photochemical quenching. It indicated that the absorbing light energy from antenna pigments in PS II is emitted as heat instead of being used for photosynthetic electron transport. When antenna pigment from PSII absorbs excess light energy, the inactivation and disruption of the photosynthetic apparatus will happen if not timely dissipated. So qN is a self-protection mechanism. There is no sharp difference in qN between *ygl7* and WT (Figure 6). This demonstrates that *ygl7* could protect the photosynthetic apparatus to a certain extent. The *ygl98* and *chl1* have not sufficient qN function. In general, the three leaf-color mutant *ygl7* has high conversion efficiency of light energy, capture efficiency of solar energy, and protection of photosynthetic apparatus. The *ygl7* is the only phenotype without influence upon grain output amongst the three mutants. Genes associated with photosynthesis were mostly up-regulated in *ygl7*. *Ygl7* possessed a higher photosynthetic efficiency in earlier physiological and biochemical studies [43]. All this data suggests that photosynthesis-related genes might be up-regulated by the accumulation of *ChlD*. The mutation of YGL7 gives the protein a new function that can put up photosynthesis-related genes.

### Differences among *ygl7*, *chl1*, *ygl3* and *ygl98* mutants

**Figure 7:**
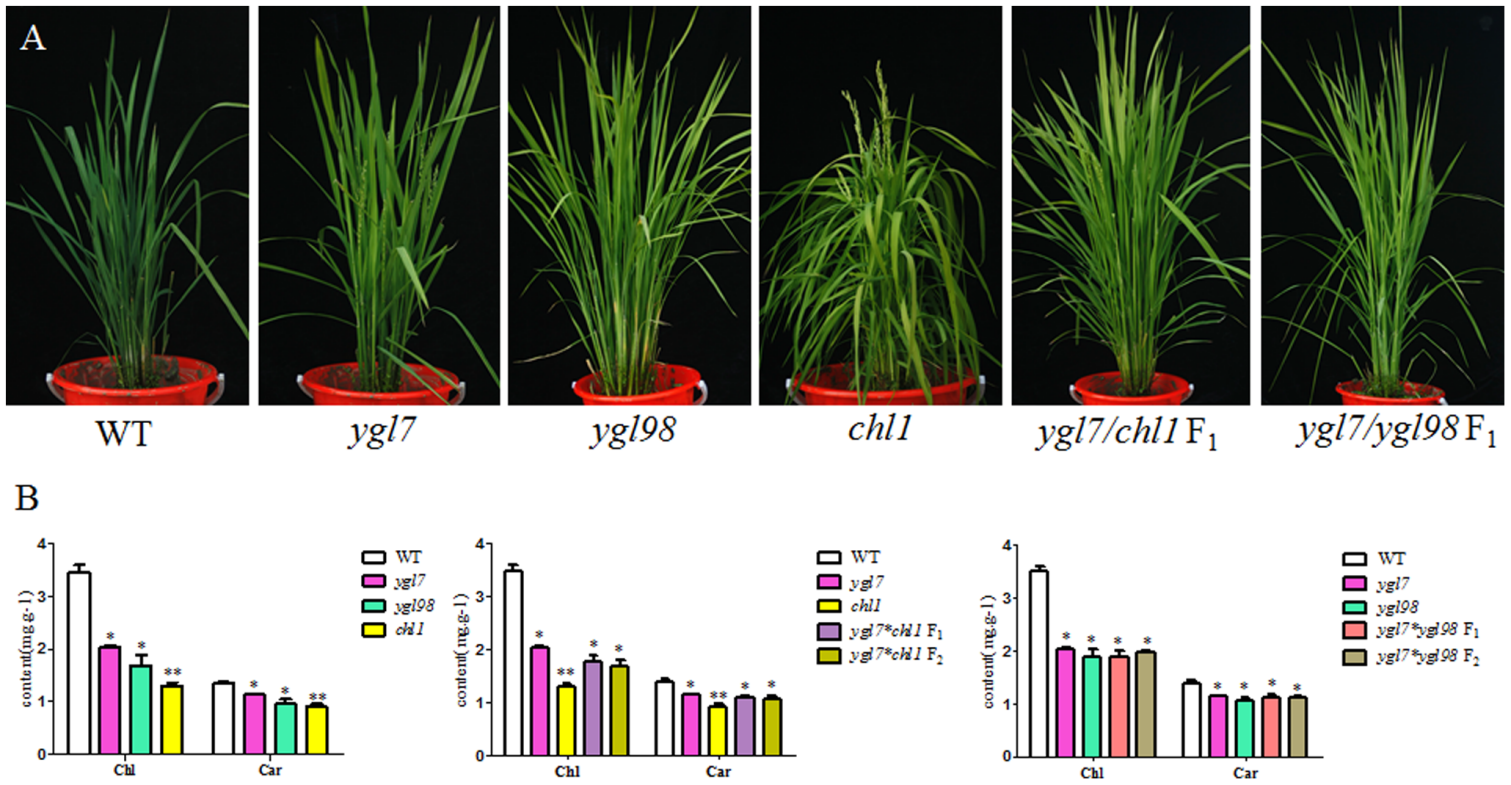
Characterization of the *OsChlD*'s alleles. A. Phenotypes of the three mutants and their crossed offspring. The *chl1* mutant is the most yellow of the three. B. Pigment contents of leaves. Values are the mean ± SD of three replicates. The *chl* has the lowest amount of pigment. All F_1_ and F_2_ offspring's pigment amounts fall between the amounts contained in their parents.

We planted the three mutants, *ygl7*, *chl1* and *ygl3*, at the field station under a standard management. In order to verify whether their mutations represent alleles, *ygl7* as the female parent was crossed with *chl1* and *ygl98*, respectively. Of the three mutants we planted under field conditions, all F_1_ and F_2_ plants had yellowy-green leaves (Figure 7, A). The content of chlorophyll and carotenoid in the F_1_ and F_2_ plants decreased to some extent (Figure 7, B). This established that the three mutants' genes are alleles. The *Chl1*'s leaf-color was the most yellow of the three mutants, while *ygl7* and *ygl98* had a yellow-green leaf-color. However, there is some discrepancy in *chl1*'s phenotype between populations in China and Korea. In our experiment, *chl1* plants had sharp yellow leaves (Hunan and Hainan Provinces). In a prior study, the *chl1* mutant displayed a yellowish-green leaf phenotype only at the seedling stage, and the abnormal leaf-color was first observed on the leaves of 2- to 3-week-old seedlings [39]. This difference will need further assessment in order to clearly understand the underlying causes.

In four alleles, *Ygl98* did not perform functional analysis [40]. *Chl1* and *Ygl3* did not perform functional complementation [9], [39]. *Ygl3*'s transgenic plants of pCAMBIA1305(35S)-RNAi exhibited the same phenotype as the *Ygl3* mutant [9]. *Chl1*'s AtChlD-KO in *Arabidopsis* (T-DNA insertional AtchlD-knockout) was a lethal mutant [39]. Similarly, our transgenic plants of pFGC5941(35S)-RNAi were lethal mutants.

## Discussion

Plants with the *ygl7* mutant maintained a yellowy-green leaf-color throughout their growth cycle, and had a sharp drop in chlorophyll content compared to WT (Figure 1). Previous studies showed that photoelectron utilization rate and photosynthetic rate are higher in *ygl7* compared with WT [43], [49]. We also found that genes associated with photosynthesis were mostly up-regulated (Figure 4; Figure 6). The net photosynthesis and dark respiration rates of *ygl7* were higher than WT [33], and the yellow-green phonotype did not negatively impact agronomic traits (Table 1). Thus, our analyses show that the *ygl7* mutant is also highly efficient in its ability to use light energy (Figure 5, 6). In addition, seeds of hybrids crossed with *ygl7* had the same grain output as the WT (Table 2). Thus, this mutant *ygl7* is an ideal leaf-color marker. Interestingly, the leaf-color of *ygl7*'s hybrids was significantly more yellow than *ygl7* itself (Figure 1). New male sterile lines show a yellow-leaf-color when the *Ygl7* gene is back-crossed to any male sterile with no associated negative agronomic traits (data not shown).

Overall, our genetic analyses of the *ygl7* mutant showed that it was controlled by a base mutation of the magnesium chelatase subunit D. Mg-chelatase plays a decisive role in the synthesis of chlorophyll because it catalyzes the insertion of magnesium into Protoporphyrin IX [50]. This enzyme consists of the three subunits I, D, and H. The three subunits take part in the synthesis of chlorophyll via a complex substance and also act independently in other metabolic pathways. The H subunit plays a role in reverse signaling to the nucleus within the chloroplast [51], controlling the SigntaE factor in cyanobacteria [52], and acts in the ABA signal transduction pathway [53]. The I subunit is the target protein of thioredoxin. The D subunit acts in exhibiting the *gun* phenotype in *Arabidopsis* [54]. OsChlD has four alleles: *Chl1* [39], *Ygl98* [40], *Ygl3* [9], and our gene *Ygl7*. The four alleles exhibit different phenotypes in the varieties in which they are found. Based on all of this evidence we can make several inferences. First, we surmise that different mutated proteins resulted in different leaf phenotypes. Second, we suggest that the D subunit may also play a role in other metabolic routes. However, these differences in leaf phenotype may simply be caused by variance in genetic backgrounds among the mutants. Of course, they may also be due to many other biological or ecological factors. A remaining question is what will happen to leaf-color when plants are grown with the same genetic background. Further study will be required to tease apart these different potential explanations.

More specific alleles continue to be identified with the continued study of rice genetics. At present, 14 genes have been shown to have alleles, amid the total of 53 cloned leaf-color genes. There has been little in-depth research on alleles, and this will be an ongoing focus of our research group. We crossed female *ygl7* with *chl1* and *ygl98* mutants. They all expressed mutated leaf-color throughout their development. However, *chl1* displayed an extreme yellowish-green leaf phenotype only at the seedling stage in Korea [39]. In China, however, *chl1* had a yellow leaf-color, while *ygl7* and *ygl98* were yellowy-green. In our study, all F_1_ and F_2_ plants had the yellowy-green leaf, and the chlorophyll and carotenoid contents of leaves taken from F_1_ and F_2_ individuals fell between the values of their two parents. Heterozygotes exhibiting an intermediate phenotypeis what is a perplexing phenomenon. It may be due to genetic neutralization, interaction of the two mutated proteins, different genetic backgrounds, or some other mechanism. Further study will address these different possibilities.

The *ygl7*-NIL is the form that represents the *ygl7* mutant's near isogenic lines. *ygl7*-NIL has the yellow leaf type instead of the yellowy-green leaf of *ygl7* (Figure 1). The same outcome was observed in offspring crossed with *ygl7*. This striking result has not been documented in previous studies of rice-leaf-color mutations. The situation is probably caused by multiple copies of the mutant gene or perhaps by an altered genetic background. We speculate that *Ygl7*'s magnesium chelatase function may be doubled by an amino acid mutation. It rapidly synthesizes magnesium protoporphyrin, but later steps in chlorophyll biosynthesis occur at normal speed thereby leading to an accumulation of magnesium protoporphyrin. Chloroplasts may send signals to the cell nucleus to regulate photosynthesis-related genes and improve the transfer rate of photoelectrons. However, feedback regulation occurs in the latter steps of chlorophyll synthesis so this may result in abnormal chlorophyll synthesis.

The fading leaf color and ultimate death of the leaves of RNAi-transformed plants (Figure 3) illustrates that rice can no longer synthesize chlorophyll once *Ygl7 is* completely silenced. Moreover, photosynthesis-related genes were mostly up-regulated in *Ygl7* (Figure 4). These phenomena show that the mutational YGL7 protein has part of OsChlD's function and promotes photosynthesis. The YGL7 protein does not lose the function of OsChlD, but as a new protein possesses a new function. There has been some research showing that accumulation of magnesium protoporphyrin can lead to reverse signal transduction and influence photoelectron transfer via regulated photosynthesis genes [55], [56]. Further research will be required to confirm that *Ygl7* plays a role in other metabolic pathways and to explore what those pathways and functions might be.

In conclusion, we have conducted a study on a yellowy-green leaf mutant of rice termed An Nong Biao 810S. This mutation was controlled by the mutational gene *Ygl7* which encodes for a magnesium-chelatase ChlD. The mutational *ygl7* had a single-nucleotide change from T to C at position 1883 bp which was located at the 12^th^ exon. The base conversion led to a change of AA (Leu 628 Ser). The mutational YGL7 protein acts with partial of OsChlD's function, and it could promote photosynthesis. The phenotype is without influence upon grain output and hybrid characteristics. The *ygl7* has a high light use efficiency, and the hybrid offspring from crosses between *yg17* and some other varieties exhibited a yellow-leaf. All these results will improve the ability to cultivate better sterile lines, facilitate hybrid seed production, and promote the purification of rice crops.

## Supporting Information

Figure S1 **The vectors in this experiment.** A. Functional complementation vector pLYL18 which reformed from pCAMBIA1300 with an ubiquitin promoter. B. RNAi vector pEGC5941.
(TIF)

Table S1 **Cloned genes that control leaf-color in rice.**
(DOC)

Table S2 **Primers used in this study.**
(DOC)

Table S3 **Polymorphic markers from screened gene-pool.**
(DOC)

Table S4 **There are 12 expressed genes at the mapping locus.**
(DOC)
