# Supplementary figures and images for: Mapped Clone and Functional Analysis of Leaf-Color Gene Ygl7 in a Rice Hybrid (Oryza sativa L. ssp. indica)
Source: PLoS One. 2014 Jun 16;9(6):e99564. doi: 10.1371/journal.pone.0099564 (PMC4059691; doi:10.1371/journal.pone.0099564)

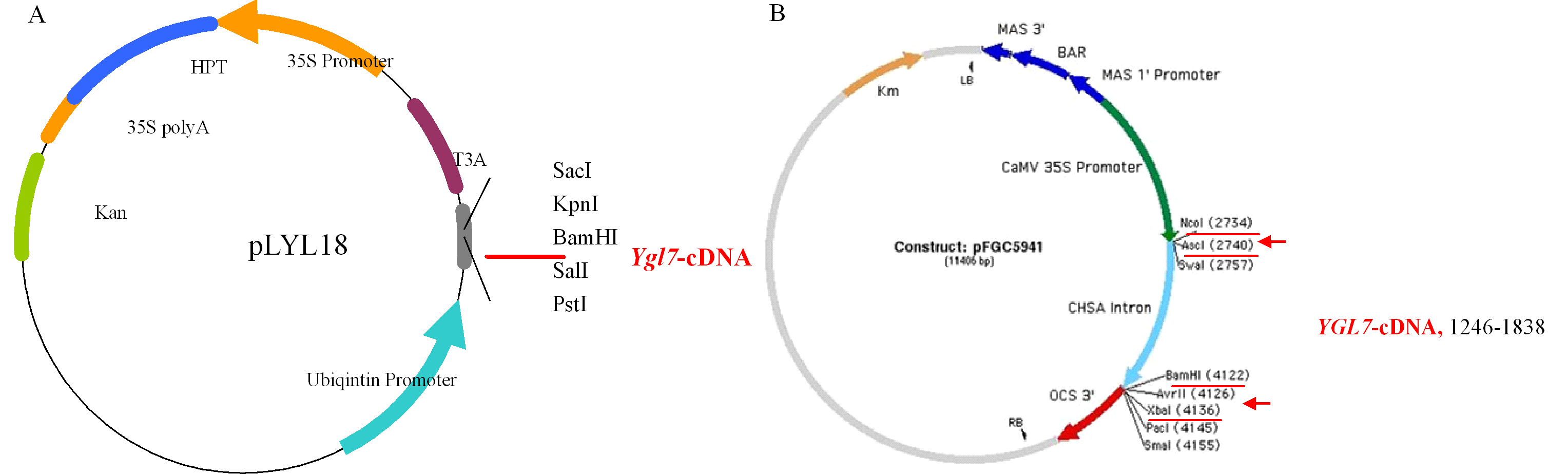

Supplement: Figure S1 — The vectors in this experiment. A. Functional complementation vector pLYL18 which reformed from pCAMBIA1300 with an ubiquitin promoter. B. RNAi vector pEGC5941. (TIF) [file pone.0099564.s001.tif]
