# Supplementary material for: Mapped Clone and Functional Analysis of Leaf-Color Gene Ygl7 in a Rice Hybrid (Oryza sativa L. ssp. indica)
Source: PLoS One. 2014 Jun 16;9(6):e99564. doi: 10.1371/journal.pone.0099564 (PMC4059691; doi:10.1371/journal.pone.0099564)
Supplement: Table S1 — Cloned genes that control leaf-color in rice. (DOC) [file pone.0099564.s002.doc]

**Table S1. Cloned genes that control leaf-color in rice.**

| Types | Genes |
| --- | --- |
| DCBC | *Chl11*(*t*) (*824ys*, *OsDVR*, alleles *590ys* and *525ys*), *Fgl* (*OsPORB*), *Lyl1-1*, *Nyc1*, *Nyc3* (*nycl1-1*, *OsNYC1*, allele *nycl1-2*), *Nol* (*nol-1*, alleles *nol-*2 and *nol-3*), *OsCAO1* (*Cbl*), *OsCAO2*, *OsCHLH*, *OsCHLI* (*Chl9*), *OsCHLD* (*Chl1*, alleles *ygl98* and *ygl3*), *OsPORA*, *Sgr* (Jiang) (allele *Sgr*(Park)), *Ygl1* |
| ICBC | *Noa1* (*OsNOA1*), *St1* (allele *Gws*), *Cdel*(*t*) (*OsGluRS*), *V3* (allele G*ra75*), *Vyl*-2007, *Gc* (*OsDET1*) |
| DDC | *Asl1*,*Cisc*(*t*), *Etl1*, *Etl2*, *Gra*(*t*), *Ita1*, *OsHAP3A*, *OsHAP3B*, *OsHAP3C*, *OsTLP27*, *OsPPR*, *Pyl-v* (*OsClpP5*), *V1* (*OsNUS1*), *V2*, *Ygl138*(*t*) , *Ysa* (*Vwl*) |
| IDC | *Adl1* (*OsClpP5*, *Adl1-1*, alleles *Adl1-2* and *Adl1-3*) , *Ntrc*, *OsCHR4*(*Oschr4-1*, alleles *Oschr4-2*, *Oschr4-3* and *Oschr4-4*) |
| CBM | *β-OsLCY* (*Phs4-1*, allele *Phs4-2*), *OsPDS*, *OsZDS* (*Phs2-1*, allele *Phs2-2*), *Z2*(alleles *OsCRTISO* (*phs3-1*,*z2-1*), *phs 3-2*, *phs* *3-3*, *Zl2*-2013 and *z2-2*) |
| ABC | *Pl* (alleles *Pl ^I^*, *Pl ^j^* and *Pl^w^*) |
| CBM | *Bgl* (*OsRopGEF10*), *OsDOS*, *GPS* (allele *NAL1*) |
| NDF | *Bgl11*(*t*) , *Grc*, *Itc, Sgra*, *Vyl-*2013 (*OsClpP6*) , *Ylc1* |

<http://www.shigen.nig.ac.jp/rice/oryzabase/genes/traitGeneClasses.jsp>,
