# Supplementary material for: Mapped Clone and Functional Analysis of Leaf-Color Gene Ygl7 in a Rice Hybrid (Oryza sativa L. ssp. indica)
Source: PLoS One. 2014 Jun 16;9(6):e99564. doi: 10.1371/journal.pone.0099564 (PMC4059691; doi:10.1371/journal.pone.0099564)
Supplement: Table S2 — Primers used in this study. (DOC) [file pone.0099564.s003.doc]

**Table S2. Primers used in this study.**

| Primer name | Sequence F (5’-3’) | Sequence R (5’-3’) | Restriction enzyme |
| --- | --- | --- | --- |
| SFP-3-6 | CTAGAGGTTGTTGCCGGTTC | ACCGCGATATTCTCACGTTC |  |
| STS3-1(AC137507) | ACAGTGTTAAGTATGTGTTCC | TAAATCCTGGGTTTCTGTGT |  |
| STS3-2(AC137507) | GATTATTGGGTAGCTAACAG | GGTTAAATTGTGGCTATTAGG |  |
| D-cDNA | TGTAAAACGACGGCCAGT | AGCGGATAACAATTTCACAGGA |  |
| D-18 F | ATATGGTACCTATGGCGATGGCCACCAC |  | *Kpn*1 |
| D-18 R | ATGCGGATCCTCACGAACTCTTCAGGTCCGA |  | *BamH*1 |
| DXJRNAiII 1 F | CGCGCCATGGCCTCCACAAGATCAAGATTCTCAAG |  | *Nco*1 |
| DXJRNAiII 1 R |  | TTGGCGCGCCGCCATTGCTATGGATCTTGAAGGTGGA | *Asc*1 |
| DXJRNAiII 2 F | CGGGATCCGCCATTGCTATGGATCTTGAAGGTGGA |  | *BamH*1 |
| DXJRNAiII 2 R |  | GCTCTAGAGCCCTCCACAAGATCAAGATTCTCAAG | *Xba*1 |
| DXJUbq | GCTCCGTGGCGGTATCAT | CGGCAGTTGACAGCCCTAG |  |
| DXJChlD | GGAAAGAGAGGGCATTAG | CAATACGATCAAGTAAGTGTT |  |
| DXJChlI | AGTAACCTTGGTGCTGTG | AATCCATCAACATTCAACTCTG |  |
| DXJChlH | CTATACATTCGCCACACT | TATCACACAACTCCCAAG |  |
| DXJJW5Rt | GAATCCCCGTCATGGGTAAAG | GCAGAAGTCGCTATGCTCCAAC |  |
| DXJYgl1 | TGGACAGTTGAAGATGTT | GAATAGGACGGTAAGGTT |  |
| DXJHema1 | CACCAGTCTGAATCATAT | CTACCACTTCTCTAATCC |  |
| DXJcab1R | AGACGTTCGCCAAGAACC | GAGGAGCTCCGGGAAGAC |  |
| DXJcab2R | GTTCTCCATGTTCGGCTTCT | GACGAAGTTGGTGGCGTAG |  |
| DXJPopa | ATCACCAAGGGCTACGTCTC | GAGTTGTTGTTCCAGCTCCA |  |
| DXJpsaA | GAGATACCACTTCCTCAT | ACTAAGAAATTCTGCGTATT |  |
| DXJpsbA | AAGTTTCTCTGATGGTATG | ATAGCACTGAATAGGGAA |  |
| DXJrbcL | GTTGAAAGGGATAAGTTGA | AATGGTTGTGAGTTTACG |  |

F, forward primers; R, reverse primers.
