# Supplementary material for: Mapped Clone and Functional Analysis of Leaf-Color Gene Ygl7 in a Rice Hybrid (Oryza sativa L. ssp. indica)
Source: PLoS One. 2014 Jun 16;9(6):e99564. doi: 10.1371/journal.pone.0099564 (PMC4059691; doi:10.1371/journal.pone.0099564)
Supplement: Table S3 — Polymorphic markers from screened gene-pool. (DOC) [file pone.0099564.s004.doc]

**Table S3. Polymorphic markers from screened gene-pool.**

| primers | No. of Chromosome | | | | | | | | | | | |
| --- | --- | --- | --- | --- | --- | --- | --- | --- | --- | --- | --- | --- |
|  | 1 | 2 | 3 | 4 | 5 | 6 | 7 | 8 | 9 | 10 | 11 | 12 |
| No. used primers | 37 | 36 | 38 | 28 | 24 | 42 | 28 | 27 | 29 | 28 | 27 | 26 |
| No. polymorphic primers | 1 | 1 | 6 | 0 | 2 | 0 | 4 | 0 | 0 | 1 | 0 | 8 |
