# Supplementary material for: Mapped Clone and Functional Analysis of Leaf-Color Gene Ygl7 in a Rice Hybrid (Oryza sativa L. ssp. indica)
Source: PLoS One. 2014 Jun 16;9(6):e99564. doi: 10.1371/journal.pone.0099564 (PMC4059691; doi:10.1371/journal.pone.0099564)
Supplement: Table S4 — There are 12 expressed genes at the mapping locus. (DOC) [file pone.0099564.s005.doc]

**Table S4. There are 12 expressed genes at the mapping locus.**

| predicted gene | predictive function | region |
| --- | --- | --- |
| Os03g59590.1 | ATP/GTP/Ca++ binding protein, putative, expressed | 33920847-33926591 |
| Os03g59600.1 | mitochondrial Rho GTPase 1, putative, expressed | 33929025-33930842 |
| Os03g59610.1 | oxidoreductase, short chain dehydrogenase/reductase family protein, putative, expressed | 33931988-33933990 |
| Os03g59620.1 | phospholipase, patatin family, putative, expressed | 33934791-33938197 |
| Os03g59630.1 | retrotransposon protein, putative, unclassified | 33945167-33946034 |
| Os03g59640.1 | magnesium-chelatase subunit chlD, chloroplast precursor, putative, expressed | 33948187-33954551 |
| Os03g59660.1 | clathrin adaptor complex small chain domain containing protein, expressed | 33962151-33964092 |
| Os03g59670.1 | basic helix-loop-helix, putative, expressed | 33966724-33968063 |
| Os03g59680.1 | PAPA-1-like conserved region family protein, expressed | 33981427-33984131 |
| Os03g59690.1 | DUF617 domain containing protein | 33987195-33988145 |
| Os03g59700.1 | peptidyl-prolyl cis-trans isomerase, putative, expressed | 33988861-33991448 |
